# Carbapenem-Resistant *Klebsiella pneumoniae* in COVID-19 Era—Challenges and Solutions

**DOI:** 10.3390/antibiotics12081285

**Published:** 2023-08-04

**Authors:** Jozef Ficik, Michal Andrezál, Hana Drahovská, Miroslav Böhmer, Tomáš Szemes, Adriána Liptáková, Lívia Slobodníková

**Affiliations:** 1 Institute of Clinical Microbiology, Central Military Hospital, 034 26 Ružomberok, Slovakia; ficikj@uvn.sk; 2 Institute of Microbiology, Faculty of Medicine, Comenius University and the University Hospital in Bratislava, 811 08 Bratislava, Slovakia; adriana.liptakova@fmed.uniba.sk; 3 Department of Molecular Biology, Faculty of Natural Sciences, Comenius University, 842 15 Bratislava, Slovakia; michal.andrezal@uniba.sk (M.A.); hana.drahovska@uniba.sk (H.D.); tomas.szemes@uniba.sk (T.S.); 4 Comenius University Science Park, 841 02 Bratislava, Slovakia; miroslav.bohmer@uniba.sk; 5 Public Health Authority of the Slovak Republic, 826 45 Bratislava, Slovakia

**Keywords:** carbapenem-resistant *Klebsiella pneumoniae*, KPC, NDM, COVID-19, antimicrobial resistance, whole genome sequencing

## Abstract

The COVID-19 era brought about new medical challenges, which, together with nosocomial bacterial infections, resulted in an enormous burden for the healthcare system. One of the most alarming nosocomial threats was carbapenem-resistant *Klebsiella pneumoniae* (CRKP). Monitoring CRKP incidence and antimicrobial resistance globally and locally is vitally important. In a retrospective study, the incidence of CRKP in the pre-COVID-19 period (2017–2019) and the COVID-19 pandemic (2020–2022) was investigated in the Central Military Hospital in Ružomberok, Slovak Republic. The relative incidence of CRKP significantly increased during the COVID-19 period—by 4.8 times, from 0.18 to 0.76%. At the same time, 47% of CRKP-positive patients also had COVID-19. Twenty-six KPC and sixty-nine NDM-producing isolates were identified. CRKPs isolated in the year 2022 were submitted to whole genome sequencing, and their susceptibility was tested to cefiderocol, ceftazidime–avibactam, imipenem–relebactam and meropenem–vaborbactam, with excellent results. KPC-producing isolates were also highly susceptible to colistin (92%). The NDM isolates revealed lower susceptibility rates, including only 57% colistin susceptibility. ST-307 prevailed in KPC and ST-11 in NDM isolates. Despite the excellent activity of new antimicrobials, rational antibiotic policy must be thoroughly followed, supported by complementary treatments and strict anti-epidemic precautions.

## 1. Introduction

The emergence and spread of carbapenem-resistant *Klebsiella pneumoniae* (CRKP) in hospitals worldwide [1,2], including the Slovak Republic [3,4], represent an urgent global public health issue, posing an enormous burden on hospital settings caring for high-risk patients [5]. Infections caused by these bacterial agents represent a significant therapeutic problem, primarily in patients in intensive care units, where bacterial superinfections increase the morbidity and mortality rates of critically ill and debilitated patients, with an overwhelming impact on hospitalisation costs [6,7]. Previous analyses show carbapenemase-producing bacteria effectively spreading during times of chaos—in war or in countries facing socio-economic and immigrant challenges [8]. A similar situation was seen in the COVID-19 era. The start of the COVID-19 pandemic brought about new healthcare challenges, with secondary bacterial infections in hospitalised patients posing additional health threats. COVID-19 patients experienced co-infection with carbapenemase producers [9]. Studies from various regions started to report increasing carbapenem resistance, dominated primarily by *Klebsiella pneumoniae* strains, occurring mainly in intensive-care units treating patients with severe COVID-19 [10,11,12,13]. The enormous increase in critically ill patients during the COVID-19 pandemic resulted in overcrowding of hospitals, especially the intensive care units, leading to an increased risk of nosocomial infections spreading. Lack of intensive care beds and mechanical ventilators also resulted in inter-hospital transfers of patients, with an additional risk of nosocomial agents dissemination as the pandemic impacted control and surveillance programs previously established against these bacteria [14]. From this aspect, the Central Military Hospital in Ružomberok (CMH), Slovak Republic, the largest military medical facility in the territory of the Slovak Republic, which provides medical care not only to professional soldiers but also to the civilian population from the entire country, belongs to the category of important medical centres threatened by the arrival of COVID-19. We set ourselves the goal of retrospectively analysing the incidence of carbapenemase-producing *Klebsiella pneumoniae* in this hospital during the COVID-19 pandemic and to compare it with the pre-COVID-19 era. Consequently, the probable factors leading to changed CRKP incidence during the COVID-19 pandemic are discussed. The antimicrobial susceptibility of the CRKP strains from the COVID-19 era is evaluated to detect the frames of antimicrobial therapy choices. Based on the results, the antibiotic therapeutic options for CRKP-caused infections in CMH during the COVID-19 pandemic are estimated, and a complementary non-antibiotic solution employing phage therapy is considered. The CRKP isolates from 2022 were submitted to whole genome sequencing (WGS) to detect the genetic determinants of virulence and antimicrobial resistance and to identify the clonal affiliation, enabling a more exact evaluation of epidemiologic relations.

## 2. Results

In a retrospective study, the carbapenem-resistant *Klebsiella pneumoniae* (CRKP) incidence and distribution were analysed from 2017 to 2022, and the pre-COVID-19 period was compared with the COVID-19 era. Clinical samples of 28,808 patients, sent for microbiologic analysis, were included. The antimicrobial susceptibility of CRKP strains isolated during the COVID-19 pandemic was examined, and the strains isolated in 2022 were submitted to whole genome sequencing. All analyses excluded multiple isolations of the same CRKP strain from the same patient.

### 2.1. The Incidence of Carbapenemase-Producing Klebsiella pneumoniae

**Figure 1:**
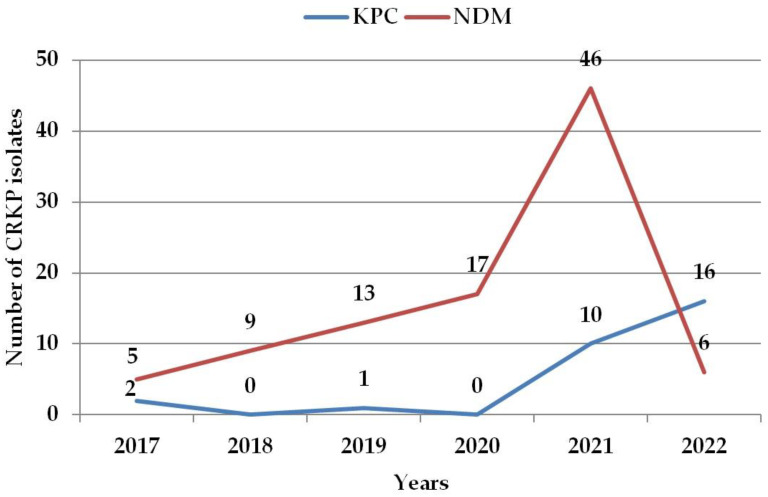
Incidence of carbapenemase-producing *Klebsiella pneumoniae* during the analysed years. CRKP—carbapenem-resistant *Klebsiella pneumoniae*; KPC—*Klebsiella pneumoniae* carbapenemase; NDM—New Delhi metallo-beta-lactamase. Note: multiple CRKP isolates from the same patient were excluded.

Clinical samples of 16,250 patients were submitted for microbiologic examination during the pre-COVID-19 period from 2017–2019. In 30 patients (0.18%), CRKP strains were isolated. In total, 3 strains produced KPC and 27 produced NDM type of carbapenemase. During the COVID-19 period (2020–2022), 12 558 patients were examined. Of them, 95 (0.76%) were CRKP-positive (for an overview, see Supplementary Tables S1 and S2). At the same time, 45 patients (47%) also had COVID-19. In total, 26 patients had KPC and 69 had NDM-carbapenemase-producing CRKP strains. The gender distribution was approximately equal, both in the basic and the CRKP-positive groups of patients (55% vs. 56.7% of females in 2017–2019; 52.6% vs. 49.5% in 2020–2022). A significant, 4.8-fold increase in CRKP relative incidence was detected during the COVID-19 period (*p* < 0.0001). NDM-producing CRKPs had a peak of their incidence in 2021, followed by a steep decrease. KPC-producing CRKPs have been slightly increasing since 2020 (Figure 1).

### 2.2. Carbapenemase-Producing Klebsiella pneumoniae Distribution in Different Age Groups, Hospital Units, and Clinical Samples

**Figure 2:**
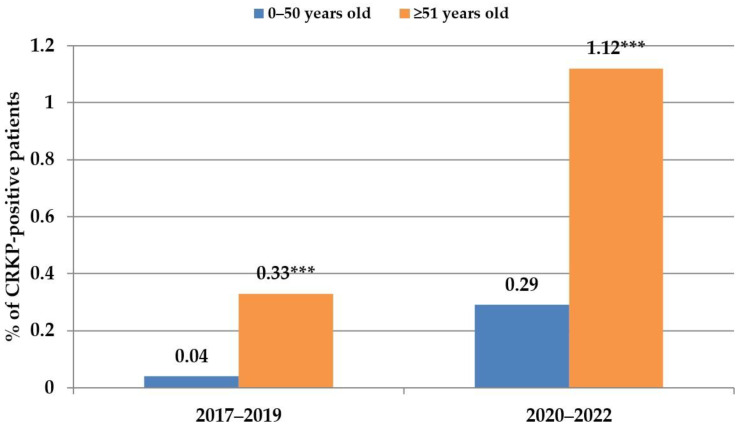
Age distribution of CRKP-positive patients with respect to risk-age for severe COVID. 0–50 years old: *p* = 0.0003; ≥51 years old: *** *p* < 0.0001.

The age of CRKP-positive patients during the COVID-19 pandemic ranged from 2 to 91 years, with a median age of 67 years (more details in Supplementary Table S1). Patients older than 50 represented the majority of CRKP-positive cases in both examined time periods. In 2017–2019, they represented 90% of all CRKP-positive cases; in 2020–2022, this proportion was 83% (Figure 2).

**Figure 3:**
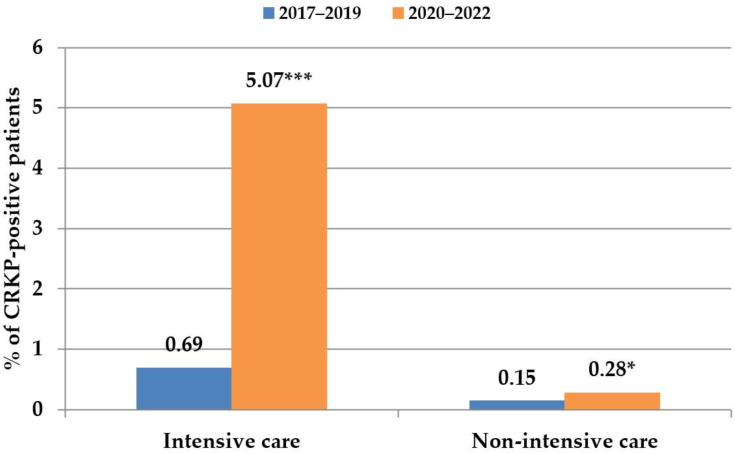
Carbapenem-resistant *Klebsiella pneumoniae* distribution in hospital units with intensive-care patients and in non-intensive-care units. * *p* < 0.05; *** *p* < 0.0001.

**Figure 4:**
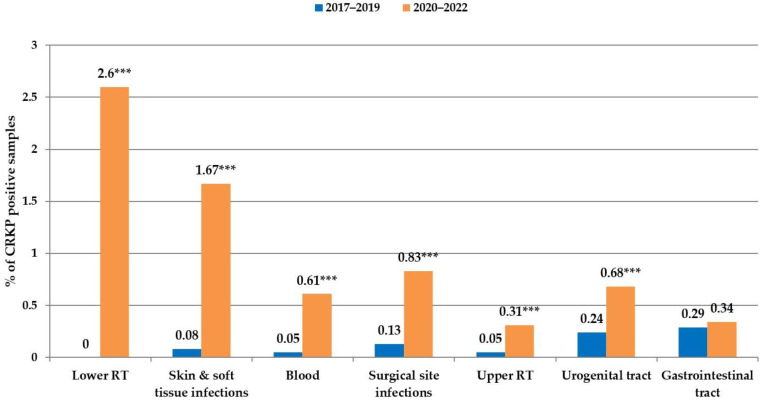
Carbapenem-resistant *Klebsiella pneumoniae* distribution in clinical samples from different body localities. *** p < 0.0001.

During the analysed time periods, the proportion of CRKPs was excessively higher in the units with intensive-care patients (Figure 3). During the COVID-19 period, in 167 patients in intensive care units, *K. pneumoniae* was isolated from the lower respiratory tract as the cause of pneumonia. In total, 58 of them had CRKP strains (38 patients were COVID-19-positive), and 48 of them suffered from CRKP-caused ventilator-associated pneumonia (VAP) (34 were COVID-19-positive). In six patients with CRKP-caused VAP, invasion of *K. pneumoniae* into the bloodstream was detected; all patients suffered from COVID. A total of 31 patients with VAP died during hospitalisation, representing nearly 65% in-hospital mortality; 24 of them were COVID-19 patients (71% mortality rate) (Supplementary Table S2). In accordance with these data, the most notable increase in CRKP-positivity during the COVID-19 pandemic was detected in samples from the lower respiratory tract (from 0 to 2.6%). They were followed by samples from skin and soft-tissue infections (from 0.08% to 1.67%) and haemocultures (from 0.05 to 0.61%) (Figure 4).

### 2.3. Antimicrobial Susceptibility of Carbapenemase-Producing Klebsiella pneumoniae

**Figure 5:**
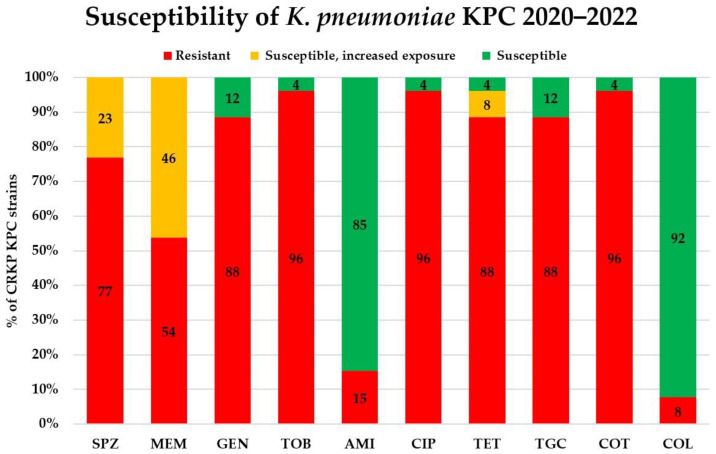
Susceptibility of KPC-producing *Klebsiella pneumoniae* isolated during COVID-19 pandemic. SPZ cefoperazone–sulbactam, MEM meropenem, GEN gentamicin, TOB tobramycin, AMI amikacin, CIP ciprofloxacin, TET tetracycline, TGC tigecycline, COT co-trimoxazole (trimethoprim–sulfamethoxazole), COL colistin. All isolates were resistant to ampicillin (intrinsic resistance); ampicillin–sulbactam, piperacillin–tazobactam, cefuroxime, cefotaxime, ceftazidime, cefepime, and ertapenem.

**Figure 6:**
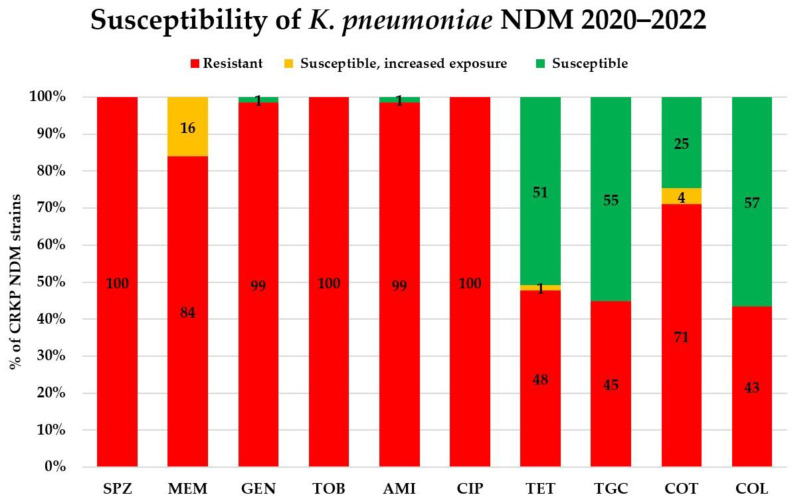
Susceptibility of NDM-producing *Klebsiella pneumoniae* isolated during the COVID-19 pandemic. SPZ cefoperazone–sulbactam, MEM meropenem, GEN gentamicin, TOB tobramycin, AMI amikacin, CIP ciprofloxacin, TET tetracycline, TGC tigecycline, COT co-trimoxazole (trimethoprim–sulfamethoxazole), COL colistin. All isolates were resistant to ampicillin (intrinsic resistance); ampicillin–sulbactam, piperacillin–tazobactam, cefuroxime, cefotaxime, ceftazidime, cefepime, and ertapenem.

Antibiotic susceptibility results were analysed in the CRKPs from the COVID-19 period. Of the 18 tested antibiotics, 8 (ampicillin—intrinsic resistance; ampicillin–sulbactam, piperacillin–tazobactam, cefuroxime, cefotaxime, ceftazidime, cefepime, and ertapenem) were ineffective. The antimicrobial susceptibility to the rest of the antibiotics considerably differed between the KPC and NDM producers (Figure 5 and Figure 6). Among the KPC-positive strains, the lowest rates of drug resistance were found for colistin (8%) and amikacin (15%), followed by gentamicin and tigecycline (both 88%). The lowest drug resistance in the NDM producers was again detected for colistin, but it was much higher than in KPC-strains: 43%. Colistin was followed by tigecycline, tetracycline, and co-trimoxazole (with 45, 48, and 71% resistance, respectively).

The distribution of MIC values for meropenem (breakpoint 2 mg/L for category “susceptible”, 8 mg/L for category “susceptible, increased exposure”), amikacin (breakpoint 8 mg/L) and colistin (breakpoint 0.2 mg/L) are shown in Supplementary Figures S1–S3. Meropenem had MICs for a comparatively large subset of KPC strains from the interval 2–8 mg/L (susceptible, increased exposure); similarly, the distribution of MIC values of colistin and amikacin in most KPC strains was closer to the breakpoint than among the NDM strains.

### 2.4. In Vitro Activity of the New Beta-Lactams

The activity of one new cephalosporin and three new combinations of beta-lactams with inhibitors of beta-lactamases was tested in the group of 12 CRKP strains isolated in the year 2022. KPC-producing isolates were tested against cefiderocol (breakpoint 2 mg/L), ceftazidime–avibactam (breakpoint 8 mg/L), imipenem–relebactam (breakpoint 2 mg/L) and meropenem–vaborbaktam (breakpoint 8 mg/L). All four therapeutics were designed to treat infections caused by multiresistant Gram-negative bacteria, including CRKPs. The NDM-producing isolates were tested only against cefiderocol, as the inhibitors of beta-lactamase combined with the other three beta-lactam drugs are inactive against class B metallo-beta-lactamases. An excellent in vitro activity was detected in all analysed isolates. Even if cefiderocol MIC values were slightly higher in the NDM-producing strains, they were considerably far from the breakpoints (Table 1 and Table 2).

### 2.5. Genome Sequencing of Selected Carbapenem-Resistant Klebsiella pneumoniae Strains

**Figure 7:**
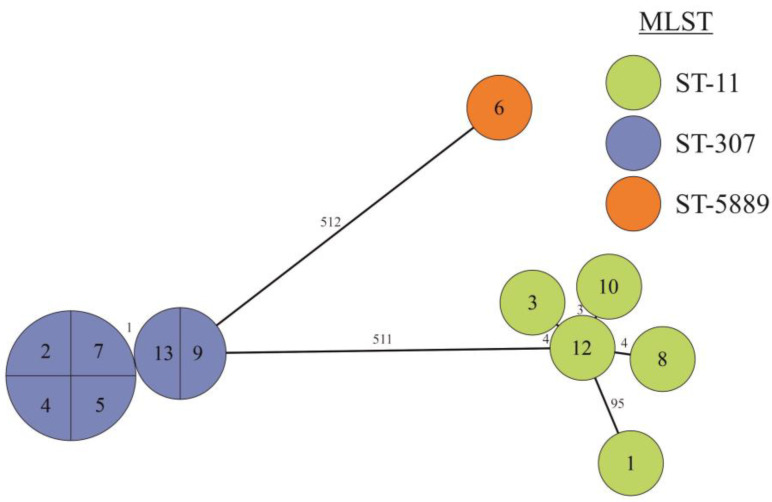
GrapeTree of 12 carbapenem-resistant *Klebsiella pneumoniae* isolates based on cgMLST. Ring sizes correspond to the number of strains with the same genotype; the designation of the strains is marked inside the ring, and the ST are designated by colour. The numbers on the lines between rings correspond to the numbers of different alleles.

Twelve CRKP isolates from 2022 were analysed via WGS. The length of the genomes ranged from 5.38 to 6.04 Mbp, and when analysed with MLST, the isolates belonged to three sequence types (STs) (Figure 7, Table 3). Five strains belonged to ST-11 of sublineage SL-258, the other six were ST-307 and SL-307, and the last strain was of the rare type ST-5889. The mutual relatedness of strains assigned to the same ST was further assessed using core genome MLST (cgMLST). Strains were clustered according to the sequence types (Figure 7). Four of the five ST-11 strains were mutually closely related as they differed by less than four alleles; the last strain was different as it possessed 95 distinct alleles and belonged to different clonal group (Supplementary Table S3). Strains of ST-307 were very homogenous, and they had almost no difference between each other. 

**Figure 8:**
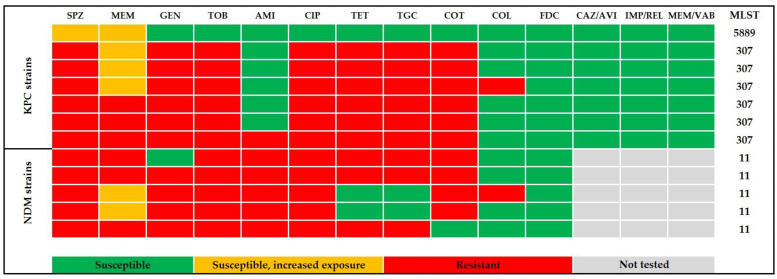
Antibiogram and the sequence-types distribution in carbapenem-resistant *Klebsiella pneumoniae* from the year 2022. SPZ cefoperazone–sulbactam, MEM meropenem, GEN gentamicin, TOB tobramycin, AMI amikacin, CIP ciprofloxacin, TET tetracycline, TGC tigecycline, COT co-trimoxazole (trimethoprim–sulfamethoxazole), COL colistin, FDC cefiderocol, CAZ/AVI ceftazidime–avibactam, IMP/REL imipenem–relebactam, MEM/VAB meropenem–vaborbaktam, KPC *Klebsiella pneumoniae* carbapenemase, NDM—New Delhi metallo-beta-lactamase. All isolates were resistant to ampicillin (intrinsic resistance); ampicillin–sulbactam, piperacillin–tazobactam, cefuroxime, cefotaxime, ceftazidime, cefepime, and ertapenem.

The presence of antibiotic resistance genes was analysed using the BV-BRC server by using the NDARO database. Five to eighteen resistance genes were detected in individual strains (Supplementary Table S4). All ST11 strains produced metallo-beta-lactamase NDM-1 and the serine beta-lactamase SHV-11; three of them also contained genes for CTX-M-15 and OXA-1. The most different ST-11 strain in cgMLST also differed in the antibiotic resistance profile. Strains belonging to ST-307 were very similar in their antibiotic resistance gene profiles. Besides KPC-2 beta-lactamase, they harboured genes for CTX-M-15, SHV-28, TEM-1, and OXA-1 beta-lactamases. The unique ST-5889 strain had the fewest resistance genes. It contained KPC-2 and SHV-1 beta-lactamases genes. The antimicrobial resistance phenotype pattern and the corresponding sequence types of the analysed carbapenemase-producing *Klebsiella pneumoniae* are shown in Figure 8.

The virulence factors in the sequenced strains were monitored using the *Klebsiella* BIGSdb database. Relatively low virulence potential was observed (Supplementary Table S5). All strains contained *mrk* operon encoding for Type 3 fimbriae and *iut*A and *glx*K genes. In addition, ST-11 strains possessed yersiniabactin genes. 

## 3. Discussion

The survey of nosocomial strains and analysis of their incidence and antimicrobial susceptibility is vital for the treatment and prevention of hospital-acquired infections. Of them, infections caused by CRKPs are of enormous importance. During the COVID-19 pandemic, increased numbers of CRKP-caused nosocomial bacterial superinfections were reported from various countries worldwide [10,11,12,13,15]. Our Central Military Hospital (CMH) was no exception: the carbapenem-resistant *Klebsiella pneumoniae* relative incidence significantly increased by 4.8 times. However, our results are in contradiction with some Chinese and Italian studies, which have not detected any statistically significant difference in the incidence ratio, colonisation rate, and infection rate of carbapenem-resistant Enterobacterales during the pandemic [16,17]. Almost half of the CRKP-positive patients of the CMH had a COVID-19 infection at the same time. 

People over 50 belong to the risk group for severe COVID-19 infection [18], and COVID-19 patients are especially threatened by secondary CRKP infection due to their extreme fragility and need for prolonged ICU stay [19]. Therefore, we tried to look at the age distribution of CRKP-positive patients. During the COVID-19 period (2020–2022), this age group represented 79% of all CRKP-positive patients. Even if CRKPs were isolated from patients over 50 with a significantly higher frequency in the COVID-19 period, similar results were obtained in the pre-COVID-19 era. In this respect, older patients may have several other individual risk factors contributing to infection caused by CRKP [20], as also may have some young patients with severe COVID-19 [19]. In our study, there were no significant differences in the incidence of CRKPs among male and female patients, in contrast with Yang et al., who detected higher CRKP proportions and resistance in male patients [16]. Similar results were presented by Mędrzycka-Dąbrowska et al. in their extensive review of CRKP infections in COVID-patients [21], who reported that most CRKP-patients were males with a mean age of 61 years.

The incidence of CRKP isolates was significantly higher in the units with intensive-care patients, in concordance with reports of many other studies; for review, see [21]. This phenomenon was even more pronounced during the COVID-19 period when conditions in the CMH changed. There were higher numbers of seriously ill patients, mainly with pneumonia, often with the need for artificial lung ventilation (ALV), which increased the risk of secondary bacterial pneumonia. In CMH, a nearly 71% mortality rate was registered among the patients with COVID-19 on ALV with CRKP-caused secondary bacterial pneumonia. Similar mortality rates were reported in a retrospective, single-centre case–control study from Spain, where carbapenemase-producing Enterobacterales were the cause of death in about two-thirds of the critically ill patients with COVID-19 [22]. The study by Dumitru et al. reports nine ICU patients with severe invasive CRKP co-infection with COVID-19. All patients but one were over 50, with considerable comorbidities. Five patients died, representing over 50% mortality rate [23]. 

Parallel with the increase in CMH patients on ALV, the CRKP-positivity of the sputum samples also increased; likewise, the Chinese study identified sputum as the primary source of CRKPs during the COVID-19 pandemic [16]. The major reservoir of *K. pneumoniae* infections is the gastrointestinal carriage [24]; however, the incidence of CRKP in gastrointestinal samples of CMH patients during the COVID-19 period increased only slightly. Artificial lung ventilation represents an increased risk of secondary nosocomial lung infection, which can be further worsened by primary viral pulmonary infection. Even if CRKP finding in the upper respiratory tract is more indicative of colonisation, it can become a source of lung invasion during intubation [25]. Nevertheless, our study revealed only a moderate increase in the incidence of CRKP in the samples from the upper respiratory tract. Several factors were revealed through analysing the potential reasons for a steep increase in the incidence of CRKP in the CMH during 2021, which primarily affected the patients of the Department of Anaesthesiology and Intensive Care Medicine (DAICM). In 2021, the number of patients with artificial lung ventilation increased four times compared to the pre-COVID-19 period. It resulted in a huge burden for the staff and a shortage of nurses and doctors. A feeling of false cleanliness could also play an important role. The staff were dressed in overalls with two gloves. Still, MDR *Klebsiella* strains can survive disinfection and remain on the overalls, allowing the spread from patient to patient, whereas there were patients from all over the Slovak Republic hospitalised in the CMH DAICM. In addition, the DAICM moved several times, for technical and organisational reasons, to different hospital areas. It is known that in such unprecedented conditions, the inability to conform completely to standard infection control practices may contribute to a shift in antimicrobial resistance, including resistance to carbapenems [17], as confirmed by the analysis of carbapenem-resistant Enterobacterales (CRE) incidence in the Italian study by Tiri et al. [15]. They detected a CRE acquisition increase from 6.7% in 2019 to 50% in March–April 2020 (the most critical COVID-19 period in Italy) and identified several factors that contributed to CRE spreading: the high intensity of care, the prone position requiring 4–5 healthcare workers equipped with personal protective equipment in a high-risk area and with extended and prolonged contact with the patient, and the presence of healthcare workers from other departments without work experience in the ICU setting. These findings were in many aspects similar to ours. Over the next year, the strain of patients in the CMH reduced, and their number decreased to half that from 2021. Moreover, the DAICM staff learned from the previous crisis. Stricter protection measures have been introduced, including wearing a disposable gown on top of the overalls during care for patients colonised by MDR strains, increased hand hygiene and extra gloves, isolation or cohortation of CRKP-positive patients (as the patient burden and the hospital capacities allowed), re-education of the staff, environmental surface monitoring for CRKP, immediate reporting of new positive cases, and increased hospital hygiene and asepsis. This resulted in a drop in NDM-producing CRKP incidence to the levels of the pre-pandemic period; however, KPC-producing CRKP appeared and started to increase slightly but steadily, even if it still did not indicate a situation of nosocomial outbreak.

CRKPs from the year 2022 were submitted to whole genome sequencing. Based on sequencing results, carbapenem-resistant *K. pneumoniae* isolated in the CMH represented a phylogenetically not very diverse group, which suggests an epidemic nosocomial spread. The majority of the isolates were of ST-11 (NDM-1 producers) and ST-307 (KPC-2 producers). Only one KPC-2 isolate was of a different ST type: ST-5889. In Europe, CRKP STs 11, 15, 101, and 258/512 are widely distributed [1]. Moreover, ST-11 is the dominant CRKP clone in China, with an alarming spread [26], successful international transmission [27], and capacity to be converted to hypervirulent (hv) carbapenemase-producing infectious agents, as demonstrated by the recently emerged ST-11 CR-HvKP strains [28]. On the other hand, CRKPs of ST-11 may also lose antimicrobial resistance and virulence [29], which means that their genome must be very plastic and requires permanent monitoring; however, this feature is typical for many other *Klebsiella pneumoniae* sequence types. *Klebsiella pneumoniae* ST-307 emerged in 2008 and, since that time, has been disseminated to different parts of the world, causing hospital outbreaks [30]. There is a lack of literary data on a unique CRKP ST-5889. Only one more isolate (from China) is deposited in the *Klebsiella* BIGSdb database. Our isolate of CRKP ST-5889 substantially differed from the other CRKPs from the CMH in a lower number of beta-lactamases and a higher susceptibility to non-beta-lactam drugs. Only one study is available concerning CRKP sequence type distribution in other hospitals in the Slovak Republic. A little broader spectrum of CRKP sequence types was present in the three medical facilities of the University Hospital in Bratislava, Slovak Republic, as reported by Koreň et al. [4]. Similar to our results, the majority of CRKPs were ST-11 NDM-1 producers. They were followed by KPC-2 producers of ST-258—a dominant ST in CRKP in Europe and America [1]—and infrequent ST-584. Just one isolate was reported from the ST-15 and ST-340, and none of our hospital’s most abundant KPC-2-producing ST-307. Our strains did not contain any genes for the production of hypermucoid capsule or siderophores other than yersiniabactin, which are important factors of hypervirulent *Klebsiella* clones [28]. This observation is in agreement with a nosocomial spread of the strains.

Treatment of patients infected with CRKP represents a big challenge. Depending on the type of produced carbapenemase, these strains are resistant to various beta-lactam drugs, and their susceptibility to non-beta-lactam drugs is not predictable due to the acquisition of different additional resistance mechanisms [31,32], as our results also documented it. Therefore, the therapeutical options are limited, and the treatment response is uncertain [33]. *Klebsiella pneumoniae* carbapenemase (KPC) and metallo-beta-lactamases, such as NDM, represent the basic molecular mechanism of CRKP resistance to carbapenems [34]. CRKPs in our study produced only these two classes of carbapenemases. Different antimicrobial resistance patterns were identified among KPC and NDM producers. The most considerable difference was in the susceptibility to amikacin, with mostly susceptible KPC producers and resistance in almost all NDM producers. Substantial differences were also detected in susceptibility to colistin—an “old” drug currently experiencing a renaissance in clinical use, driven by the increased spreading of multidrug-resistant Gram-negative rods in hospital settings. Even if rather toxic, colistin was considered, in many cases, a last-resort drug. Due to the spread of antimicrobial resistance and the appearance of CRKP, it is now often used as a first-line treatment for infections caused by these bacteria [35]. With more and more frequent use of colistin in therapy, colistin-resistant strains with modified LPS molecules have started to emerge [36]. In our study, the KPC-producing CRKP strains had only a low colistin-resistance rate; however, we did not expect such high colistin resistance in the NDM producers (over 40%). Also, the colistin-MIC distribution was less favourable for NDM-strains, with values far from the breakpoint, which may be a disadvantage when a combination treatment is considered. Growing numbers of colistin-resistant CRKP isolates were also reported from University Hospital facilities in the Slovak Republic, even if not so alarming—from 0% published in 2019 to 2.5% in 2022 [3,4]. When analysing this extremely high colistin-resistance rate in the CMH among the NDM CRKP strains, the most probable reason is the epidemic spread of the same colistin-resistant NDM *Klebsiella pneumoniae* strain during the COVID-19 period (as discussed above). Based on in vitro studies [37] and clinical trials [38], colistin in combination with either meropenem or amikacin could be a valid therapeutic option against colistin-resistant CRKP isolates. Unfortunately, this option could not have been applied in most CMH patients with NDM-producing CRKPs due to their high resistance rate against these antimicrobial drugs. Tigecycline is another drug with activity against CRKP and a positive effect on the survival of patients with severe CRKP-caused infections when applied in high doses in combination therapy [39]. However, resistance emerging has been described during the treatment of CRKP infections [40]. In our study, based on the susceptibility test results, the successful effect of the combination therapy with tigecycline could be expected in more than half of the infections caused by NDM-producing CRKPs, but only in less than 20% of cases caused by KPC-producers. The unusual combination of ertapenem with another carbapenem in the therapy of CRKP-caused infections is based on the evidence that ertapenem could bind to the carbapenemase in a very high affinity which prevents the hydrolysis of the other carbapenem molecule and preserves its bactericidal activity [41]. From meropenem’s MIC distributions, we guess this therapeutical option could work better for KPC producers, which had MICs distribution closer to the breakpoint.

Correct identification of carbapenemase type is crucial for selecting the most suitable antimicrobial agent among those recently introduced to the therapeutic practice [34]. In general, CRKPs, which produce metallo-beta-lactamase (including NDM), are resistant to all beta-lactams (and their combinations with beta-lactamase inhibitors), except cefiderocol—a first siderophore cephalosporin—and the monobactam aztreonam [42]. In metallo-beta-lactamase-producing CRKP, aztreonam can frequently be inactivated by other beta-lactamases. A combination of ceftazidime–avibactam and aztreonam can have a synergistic effect, as avibactam can inactivate other beta-lactamases to preserve aztreonam activity [43,44]. However, testing of these two antimicrobials was not included in our CRKP strains’ routine susceptibility testing scheme.

For therapy of infections caused by KPC producers, combinations of beta-lactams with new beta-lactamase inhibitors, such as ceftazidime–avibactam, meropenem–vaborbactam, or imipenem–relebactam, or a new cefalosporin, cefiderocol, are suggested [45]. These new drugs are still only seldom used in the Slovak Republic. Their rare use is in accordance with the excellent susceptibility detected in CRKPs isolated in 2022 in CMH. Unfortunately, there are already reports on resistance emergence against these new valuable drugs [45,46]. Clinical failure of treatment with ceftazidime–avibactam was described by Dumitru et al. [25] in patients whose diagnosis was late. Solutions may be the early and correct identification of the CRKP-produced carbapenemase type, early therapy with effective drugs, strict adherence to rational antimicrobial therapy, and a judicious prescription of these valuable antimicrobials. These precautions may slow down the emergence and spread of resistant strains and preserve the activity of the drugs mentioned above for the future [45,46]. Alternative or complementary therapies—such as phage therapy—can also be helpful in these efforts. Several reports of COVID-19 patients’ treatment with therapeutic phages were published during the COVID-19 pandemic. COVID-19 patients with secondary bacterial infections were successfully treated with bacteriophages in China [47] and Russia [48], and the FDA has also allowed the use of phage therapy for COVID-19 patients who are in a critical state [49]. This old and nowadays rediscovered antibacterial therapy has many advantages over traditional antibiotics. Phages are highly specific to their hosts, preserving the microbiota equilibrium during treatment. In the infectious focus, they amplify and may spread and attack their specific hosts over the body without respect to antimicrobial resistance mechanisms and do not select for antimicrobial resistance [50]. Phage therapy is usually safe, but the phage-induced release of endotoxins may lead to an immune reaction, depending on the phages, delivery routes, or host immune status, as it was observed by Wu et al. (2022). One of their COVID-19 patients developed transient fever and IL-6 and IL-8 storm four hours after phage inhalation; such reaction might be a result of triggering human immune responses directly by antigens of therapeutic phages or indirectly via the rapid lysis of the targeted bacteria [47].

## 4. Materials and Methods

### 4.1. Data Resources

The laboratory information system database of the Institute of Clinical Microbiology, Central Military Hospital in Ružomberok, Slovak Republic, was analysed for data on *Klebsiella pneumoniae* isolations during 2017–2022. The incidence of CRKP strains in the COVID-19 and pre-COVID-19 periods (years 2020–2022 and 2017–2019, respectively) and the antimicrobial susceptibility of CRKP strains during the COVID-19 period were then retrospectively analysed. Data on CRKP strains distribution among patients, hospital units, and biological sample types were also examined. Multiple isolations of the same strain from the same patient were excluded from the analysis. The patients’ personal data protection, in conformity with the GDPR, was applied during the analysis.

### 4.2. Bacterial Strains Isolation, Identification and Long-Term Preservation

The clinical samples were collected, transported, and processed in conformity with the standard diagnostic procedures. The identification of *Klebsiella pneumoniae* strains was carried out using the biochemical ENTEROtest 16 kit (Erba Lachema s.r.o., Brno, Czech Republic). Ambiguous results were confirmed using MALDI-TOF MS. All CRKPs isolated during 2022 were long-term preserved by freezing in cryoprotective medium (5% *w*/*v* Skim Milk, Biolife, Milan, Italy) at −20 °C. 

### 4.3. Antimicrobial Susceptibility Testing and Carbapenemase Type Detection

A colourimetric micro-method for automated susceptibility testing MIDITECH (Bell Novaman, Bratislava, Slovak Republic), meeting the antimicrobial susceptibility testing recommendations of the CLSI [51], was used for susceptibility testing to 18 antimicrobial agents (ampicillin, ampicillin–sulbactam, piperacillin–tazobactam, cefuroxime, cefotaxime, ceftazidime, cefoperazone–sulbactam, cefepime, ertapenem, meropenem, gentamicin, tobramycin, amikacin, tigecycline, ciprofloxacin, tetracycline, colistin, and co-trimoxazole) and 2 cephalosporin combinations with inhibitors of beta-lactamase for detection of extended-spectrum beta-lactamase production (cefotaxime-clavulanate, ceftazidime-clavulanate). E-test (Liofilchem, S.r.l., Roseto degli Abruzzi, Italy) was used to detect the susceptibility to cefiderocol, imipenem–relebactam, ceftazidime–avibactam, and meropenem–vaborbactam. MIC values were interpreted according to the EUCAST clinical breakpoints [52]. Screening of carbapenemase-positive strains and detection of carbapenemase production (using chromogenic pH metric Carba NP test; Diagnostics, s.r.o., Galanta, Slovak Republic) was performed according to EUCAST guidelines [53]. The rapid phenotypic identification of the carbapenemase type was made via immunochromatographic CARBA-5 assay (NG-Biotech Laboratoires, Guipry-Messac, France), designed for the detection of five major carbapenemases types (KPC, NDM, VIM, IMP, and OXA-48). Respecting the guidelines of the Ministry of Health of the Slovak Republic, the carbapenemase-producing *Klebsiella pneumoniae* strains were sent to the National Reference Centre for Antimicrobial Resistance Surveillance for confirmation.

### 4.4. Genome Analysing of Klebsiella pneumoniae Isolates

Overnight bacterial cultures grown on Luria–Bertani (LB) medium at 37 °C were used. DNA was isolated using the Higher PurityTM Bacterial Genomic DNA Isolation Kit (CanvaxBiotech; Cordoba, Spain) and was quantified using the Qubit ds-DNA HS Assay Kit (Thermofisher Scientific; Eugene, OR, USA). Sequencing libraries were preprepared using the Nextera XT DNA Library Prep Kit protocol (Illumina; San Diego, CA, USA) and purified on AMPure XP magnetic beads (Beckman Coulter Life Sciences; Indianapolis, IN, USA). The quality of the library was checked using a high-sensitivity DNA electrophoresis chip and a 2100 Bioanalyzer Instrument (Agilent Technologies; Santa Clara, CA, USA). Sequencing was carried out with 2 × 150 bp reads using the Illumina NextSeq 500 platform (Illumina). The obtained sequencing data were assembled de novo with SPAdes using a standard setting of parameters (Center for Algorithmic Biotechnology; St. Petersburg State University, St Petersburg, Russia) [54] and annotated using the online BV-BRC program (Bacterial and Viral Bioinformatics Resource Center, https://www.bv-brc.org (accessed on 14 July 2023). The criterion of 100% identity and 100% coverage with the reference gene was used as the threshold for gene presence. The genomes were further analysed using the CGE database (Center for Genomic Epidemiology; Kongens, Lyngby, Denmark), the BV-BRC server, and the BIGSdb database (Institut Pasteur MLST, Paris, France). The standard MLST and cgMLST based on 629 genes available in the *Klebsiella* BIGSdb database (https://bigsdb.pasteur.fr/klebsiella/, (accessed on 16 July 2023) were used for the determination of strain relatedness. GrapeTree was used to visualise strain clusters based on the *Klebsiella* cgMLST analysis. The sequenced genomes were deposited in the NCBI database under bioproject accession number PRJNA996751 and into the BIGSdb-Pasteur *Klebsiella pneumoniae* database under accession numbers 57223–57235. 

### 4.5. Statistical Analysis

The results were statistically analysed with the chi-square test using Excel software version 2019 (Microsoft Corporation, Redmond, WA, USA). *p* values of <0.05 were considered statistically significant.

## 5. Conclusions

Isolates of CRKP increased in the CMH during the COVID-19 pandemic. The molecular mechanisms responsible for carbapenem resistance were KPC and NDM carbapenemase production. The recent isolates had relatively low phylogenetic diversity, which may indicate an epidemical spread; all were susceptible to new antimicrobials appointed to treat CRKP-caused infections. Despite the excellent activity of new antimicrobials, a rational antibiotic policy must be thoroughly followed, supported by complementary treatments and strict anti-epidemic precautions. Based on the available literary data, bacteriophages, with their high specificity and rare side effects, would be of great value in the therapy of nosocomial bacterial superinfections caused by CRKP strains, and not only in COVID-19 patients.

## Figures and Tables

**Table 1:** Ceftazidime–avibactam, imipenem–relebactam and meropenem–vaborbaktam in vitro activity against KPC-producing carbapenem-resistant *Klebsiella pneumoniae*.

Ceftazidime–Avibactam MIC [mg/L]	KPC (n)	Imipenem–Relebactam MIC [mg/L]	KPC (n)	Meropenem–Vaborbaktam MIC [mg/L]	KPC (n)
0.032	1	0.094	1	0.023	3
0.094	1	0.125	2	0.032	3
0.190	2	0.190	1	0.047	1
0.380	1	0.250	1		
0.750	2	0.380	1		
		0.5	1		

MIC—minimal inhibitory concentration; KPC—*Klebsiella pneumoniae* carbapenemase.

**Table 2:** Cefiderocol in vitro activity against carbapenem-resistant *Klebsiella pneumoniae*.

Cefiderocol MIC [mg/L]	KPC (n)	NDM (n)
0.016	1	-
0.032	4	-
0.064	1	1
0.094	1	1
0.19	-	2
0.25	-	1

MIC—minimal inhibitory concentration; KPC—*Klebsiella pneumoniae* carbapenemase; NDM—New Delhi metallo-beta-lactamase.

**Table 3:** Characterisation of 12 recent isolates of carbapenem-resistant *Klebsiella pneumoniae* with sequenced genomes.

Sublineage	ST	Clonal Group	Serotype (wzi) ^1^	Carbapenemase Gene	No. of Strains
SL-258	11	CG340	K15:O4 (50)	NDM-1	4
		CG3666	? (24)	NDM-1	1
SL-307	307	CG307	K2:O1/2 (173)	KPC-2	6
SL-10716	5889	CG11340	K14/15:O3 (new)	KPC-2	1

^1^ Serotype determined according to K-PAM (*wzi* gene allele number).

## Data Availability

The data for this study have been deposited in the NCBI database under bioproject accession number PRJNA996751 and into The BIGSdb-Pasteur *Klebsiella pneumoniae* database under accession numbers 57223–57235.

## Supplementary Materials

The following supporting information can be downloaded at: https://www.mdpi.com/article/10.3390/antibiotics12081285/s1, Figure S1: Meropenem MIC values distribution in KPC (a) and NDM (b) producing carbapenemase-resistant *Klebsiella pneumoniae;* Figure S2: Amikacin MIC values distribution in KPC (a) and NDM (b) producing carbapenemase-resistant *Klebsiella pneumoniae;* Figure S3: Colistin MIC values distribution in KPC (a) and NDM (b) producing carbapenemase-resistant *Klebsiella pneumoniae;* Table S1: Distribution of patients with carbapenem-resistant *Klebsiella pneumoniae* during COVID-19 pandemic to age categories; Table S2: Patients infected by carbapenem-resistant *Klebsiella pneumoniae* during the COVID-19 pandemic; Table S3: Taxonomic characterization of sequenced *Klebsiella pneumoniae* strains; Table S4: Genes encoding antibiotic resistance in sequenced *Klebsiella pneumoniae* strains; Table S5: Virulence factor genes in sequenced *Klebsiella pneumoniae* strains.
