# Supplementary material for: Carbapenem-Resistant Klebsiella pneumoniae in COVID-19 Era—Challenges and Solutions
Source: Antibiotics (Basel). 2023 Aug 4;12(8):1285. doi: 10.3390/antibiotics12081285 (PMC10451955; doi:10.3390/antibiotics12081285)
Supplement: Supplementary file 1 [file antibiotics-12-01285-s001.zip › Supplementary Table S1.pdf]

Table S1 Distribution of patients with carbapenem-resistant *Klebsiella pneumoniae* during the COVID-19 pandemic to age categories

Age groups	All patients / COVID-19 positive (n)	Males: All / COVID-19 positive (n)	Females: All / COVID-19 positive (n)
**0-5**	1 / 0	0 / 0	1 / 0
**6-17**	0	0	0
**18-49**	14 / 4	8 / 3	6 / 1
**50-64**	28 / 16	17 / 10	11 / 6
**≥65**	52 / 24	31 / 14	21 / 10
