# Supplementary material for: Carbapenem-Resistant Klebsiella pneumoniae in COVID-19 Era—Challenges and Solutions
Source: Antibiotics (Basel). 2023 Aug 4;12(8):1285. doi: 10.3390/antibiotics12081285 (PMC10451955; doi:10.3390/antibiotics12081285)
Supplement: Supplementary file 1 [file antibiotics-12-01285-s001.zip › Supplementary Table S2.pdf]

Table S2 Patients infected with carbapenem-resistant *Klebsiella pneumoniae* during the COVID-19 pandemic

	All patients / COVID-19 positive		
	**Males (n)**	**Females (n)**	**Total (n)**
**All CRKP-positive patients (2020-2022)**	48 / 28	47 / 17	95 / 45
**Patients in intensive care**	34 / 22	24 / 16	58 / 38
**Patients on APV**	30 / 19	18 / 15	48 / 34
**Patients with CRKP-caused VAP**	30 / 19	18 / 15	48 / 34
**CRKP bloodstream invasion in patients with pneumonia**	3 / 3	3 / 3	6 / 6
**In-hospital mortality – all patients in intensive care**	29 / 19	18 / 14	47 / 33
**In-hospital mortality - patients with CRKP-caused VAP**	18 / 12	13 / 12	31 / 24

CRKP – carbapenem-resistant Klebsiella pneumoniae, APV - artificial pulmonary ventilation; VAP – ventilator-associated pneumonia
