# Supplementary material for: Carbapenem-Resistant Klebsiella pneumoniae in COVID-19 Era—Challenges and Solutions
Source: Antibiotics (Basel). 2023 Aug 4;12(8):1285. doi: 10.3390/antibiotics12081285 (PMC10451955; doi:10.3390/antibiotics12081285)
Supplement: Supplementary file 1 [file antibiotics-12-01285-s001.zip › Supplementary Table S3.pdf]

Table S3 Taxonomic characterization of sequenced *Klebsiella pneumoniae* strains

Strain	Alternative name	Sublineage	Clonal group	ST	Serotype (wzi)[1]	MLST id
6	KMB-1239	SL10716	CG11340	5889	K14/15:O3 (new)	57228
5	KMB-1238	SL307	CG307	307	K2:O1/2 (173)	57227
7	KMB-1240	SL307	CG307	307	K2:O1/2 (173)	57229
9	KMB-1242	SL307	CG307	307	K2:O1/2 (173)	57231
4	KMB-1237	SL307	CG307	307	K2:O1/2 (173)	57226
13	KMB-1246	SL307	CG307	307	K2:O1/2 (173)	57235
2	KMB-1235	SL307	CG307	307	K2:O1/2 (173)	57224
1	KMB-1234	SL258	CG3666	11	? (24)	57223
3	KMB-1236	SL258	CG340	11	K15:O4 (50)	57225
8	KMB-1241	SL258	CG340	11	K15:O4 (50)	57230
10	KMB-1243	SL258	CG340	11	K15:O4 (50)	57232
12	KMB-1245	SL258	CG340	11	K15:O4 (50)	57234

[1] Serotype determined according to K-PAM (wzi gene allele number)
