# Supplementary material for: Carbapenem-Resistant Klebsiella pneumoniae in COVID-19 Era—Challenges and Solutions
Source: Antibiotics (Basel). 2023 Aug 4;12(8):1285. doi: 10.3390/antibiotics12081285 (PMC10451955; doi:10.3390/antibiotics12081285)
Supplement: Supplementary file 1 [file antibiotics-12-01285-s001.zip › Supplementary Table S4.pdf]

Table S4 Genes encoding antibiotic resistance in sequenced *Klebsiella pneumoniae* strains

									**Aminoglycosides**	**Bleomycine**									**Betalactams**	**Trimetoprim**		**Sulfonamids**	**Phosphomycine**		**Chloramphenicol**		**Chloramfenikol/ florfenickol**	**Chinolones**	**Rifampin**					**Eflux pumps**	**Q amonium com**	**Tetracyclines**
**Strain**	**MLST**	AadA2	Arr-2	APH(3'')-Ib	APH(6)-Id	AAC(3)-IIa	AAC(6')-Ib	AAC(6')-Ib4	RmtF1	Ble-MBL	TEM-156	CTX-M-15	SHV-1	SHV-11	SHV-28	TEM-1	OXA-1	**KPC-2**	**NDM-1**	DfrA12	Sul1	Sul2	FosA	CatB	CatB3*	FloR	Mph(A)	QnrB1	Arr-2	OqxA	OqxB	OqxB19	OqxB20	OqxB25	QacE delta	Tet(A)
6	5889												+					+					+							+				+		
5	307			+	+	+						+			+	+	+	+				+	+		+			+		+		+				+
7	307			+	+	+						+			+	+	+	+				+	+		+			+		+		+				+
9	307			+	+	+						+			+	+	+	+				+	+		+			+		+		+				+
4	307			+	+	+						+			+	+	+	+				+	+		+			+		+		+				+
13	307			+	+	+						+			+	+	+	+				+	+		+			+		+		+				+
2	307			+	+	+						+			+	+	+	+				+	+		+			+		+		+				+
1	11									+				+		+			+			+	+			+	+			+			+			
3	11	+					+		+	+				+					+	+	+		+	+						+	+				+	
8	11	+				+		+	+	+		+		+			+		+	+	+		+	+	+				+	+	+				+	
10	11	+				+			+	+		+		+			+		+	+	+		+	+	+				+	+	+				+	
12	11		+				+		+	+				+					+				+	+						+	+					
