# Supplementary material for: Carbapenem-Resistant Klebsiella pneumoniae in COVID-19 Era—Challenges and Solutions
Source: Antibiotics (Basel). 2023 Aug 4;12(8):1285. doi: 10.3390/antibiotics12081285 (PMC10451955; doi:10.3390/antibiotics12081285)
Supplement: Supplementary file 1 [file antibiotics-12-01285-s001.zip › Supplementary Table S5.pdf]

Table S5 Virulence factor genes in sequenced *Klebsiella pneumoniae* strains

		**Type 3 fimbriae**								**Yersiniabactin**											**Aerobactin**	**Glycerate kinase**
**Strain**	**MLST**	mrkA	mrkB	mrkC	mrkD	mrkF	mrkH	mrkI	mrkJ	ybtA	ybtE	ybtP	ybtQ	ybtS	ybtT	ybtU	ybtX	irp1	irp2	fyuA	iutA	glxK
6	5889	+	+	+	+	+	+	+	+												+	+
5	307	+	+	+	+	+	+	+	+												+	+
7	307	+	+	+	+	+	+	+	+												+	+
9	307	+	+	+	+	+	+	+	+												+	+
4	307	+	+	+	+	+	+	+	+												+	+
13	307	+	+	+	+	+	+	+	+												+	+
2	307	+	+	+	+	+	+	+	+												+	+
1	11	+	+	+	+	+	+	+	+	+	+	+	+	+	+	+	+	+	+	+	+	+
3	11	+	+	+	+	+	+	+	+	+	+	+	+	+	+	+	+	+	+	+	+	+
8	11	+	+	+	+	+	+	+	+	+	+	+	+	+	+	+	+	+	+	+	+	+
10	11	+	+	+	+	+	+	+	+	+	+	+	+	+	+	+	+	+	+	+	+	+
12	11	+	+	+	+	+	+	+	+	+	+	+	+	+	+	+	+	+	+	+	+	+
