# Supplementary figures and images for: Carbapenem-Resistant Klebsiella pneumoniae in COVID-19 Era—Challenges and Solutions
Source: Antibiotics (Basel). 2023 Aug 4;12(8):1285. doi: 10.3390/antibiotics12081285 (PMC10451955; doi:10.3390/antibiotics12081285)

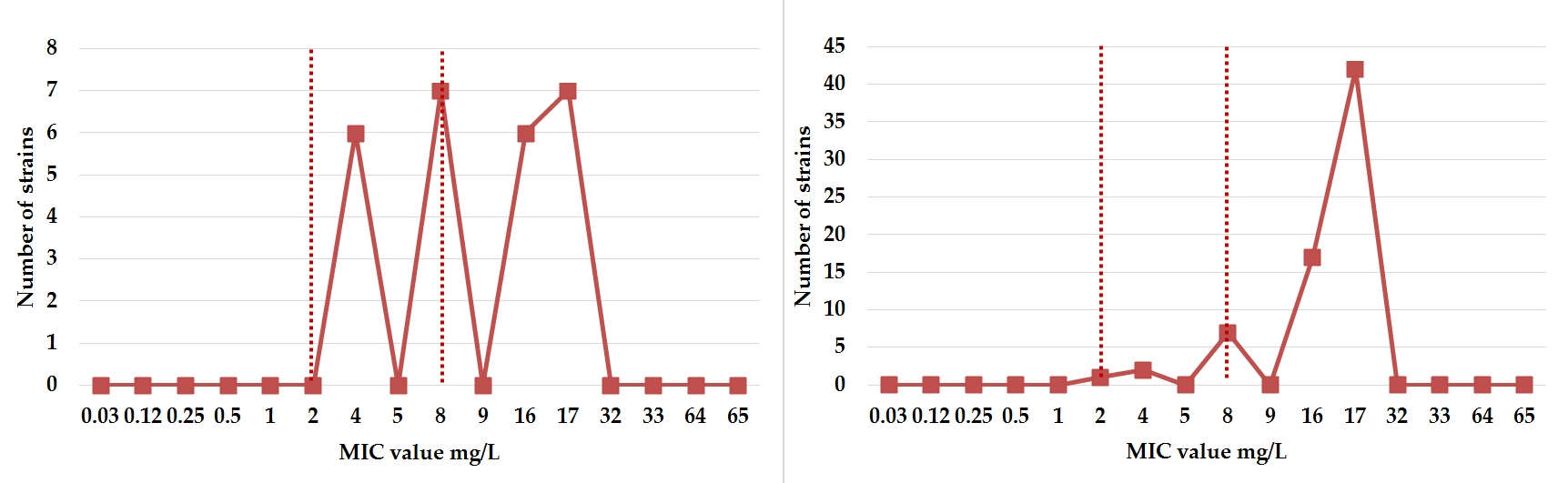

Supplement: Supplementary file 1 [file antibiotics-12-01285-s001.zip › Figure S1. Meropenem MIC values distribution in KPC (a) and NDM (b) producing carbapenem-resistant Klebsiella pneumoniae.jpg]

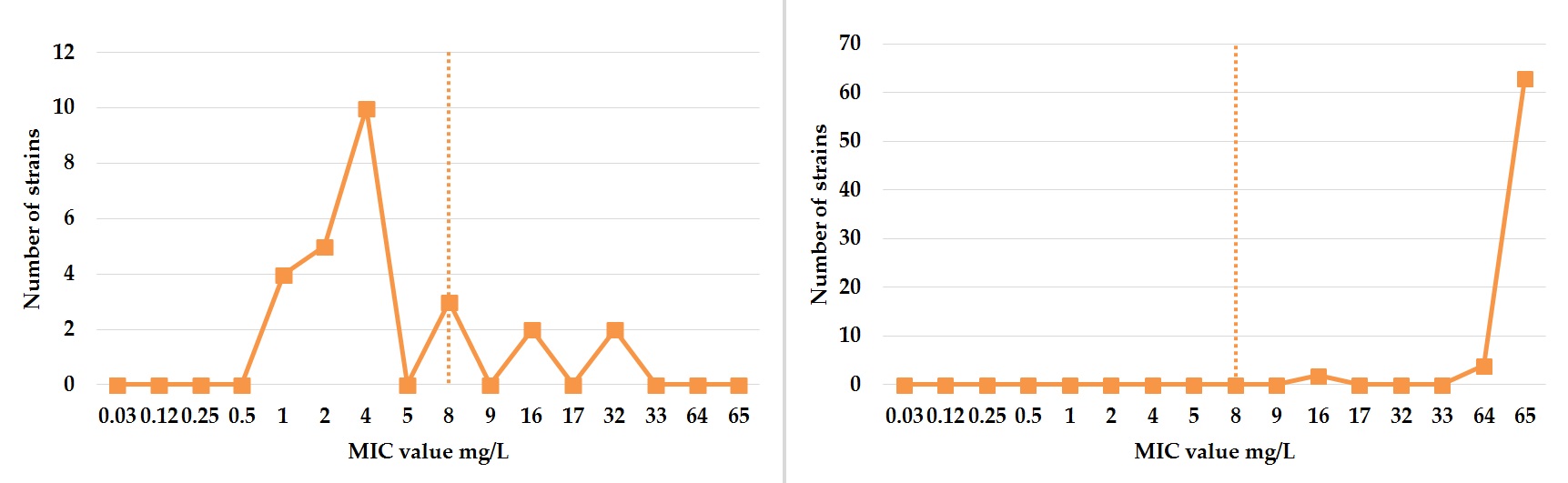

Supplement: Supplementary file 1 [file antibiotics-12-01285-s001.zip › Figure S2. Amikacin MIC values distribution in KPC (a) and NDM (b) producing carbapenem-resistant Klebsiella pneumoniae.jpg]

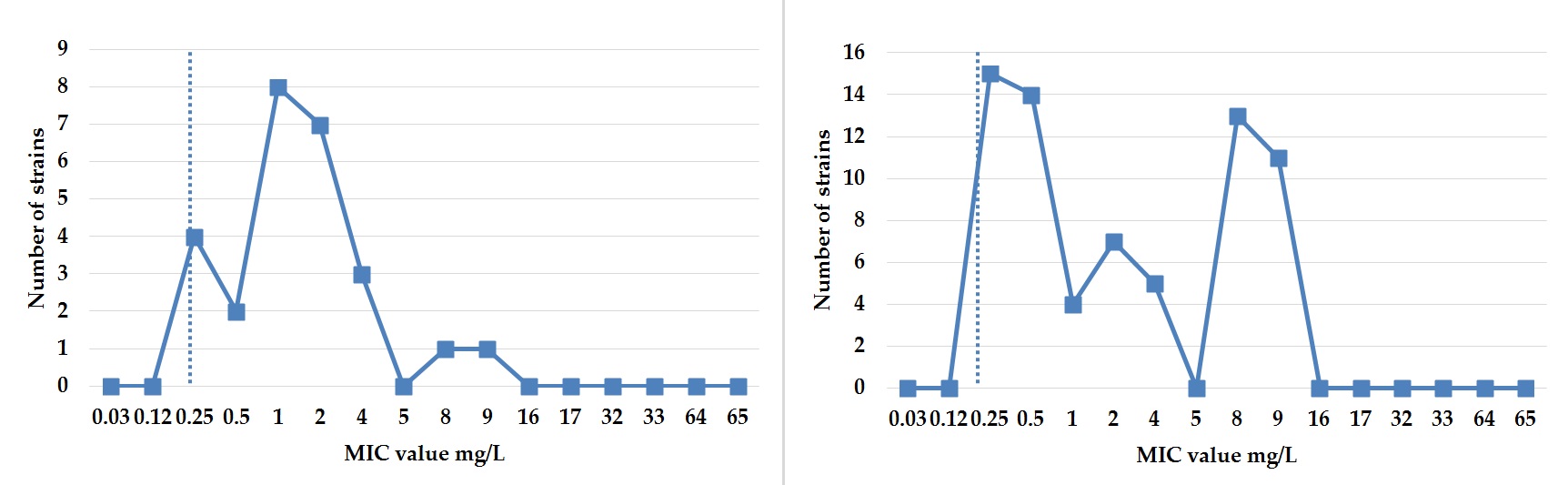

Supplement: Supplementary file 1 [file antibiotics-12-01285-s001.zip › Figure S3. Colistin MIC values distribution in KPC (a) and NDM (b) producing carbapenem-resistant Klebsiella pneumoniae.jpg]
